# Process intensification through microbial strain evolution: mixed glucose-xylose fermentation in wheat straw hydrolyzates by three generations of recombinant *Saccharomyces cerevisiae*

**DOI:** 10.1186/1754-6834-7-49

**Published:** 2014-04-03

**Authors:** Vera Novy, Stefan Krahulec, Manfred Wegleiter, Gerdt Müller, Karin Longus, Mario Klimacek, Bernd Nidetzky

**Affiliations:** 1 Institute of Biotechnology and Biochemical Engineering, Graz University of Technology, Petersgasse 12/I, 8010 Graz, Austria

## Abstract

**Background:**

Lignocellulose hydrolyzates present difficult substrates for ethanol production by the most commonly applied microorganism in the fermentation industries, *Saccharomyces cerevisiae*. High resistance towards inhibitors released during pretreatment and hydrolysis of the feedstock as well as efficient utilization of hexose and pentose sugars constitute major challenges in the development of *S. cerevisiae* strains for biomass-to-ethanol processes. Metabolic engineering and laboratory evolution are applied, alone and in combination, to adduce desired strain properties. However, physiological requirements for robust performance of *S. cerevisiae* in the conversion of lignocellulose hydrolyzates are not well understood. The herein presented *S. cerevisiae* strains IBB10A02 and IBB10B05 are descendants of strain BP10001, which was previously derived from the widely used strain CEN.PK 113-5D through introduction of a largely redox-neutral oxidoreductive xylose assimilation pathway. The IBB strains were obtained by a two-step laboratory evolution that selected for fast xylose fermentation in combination with anaerobic growth before (IBB10A02) and after adaption in repeated xylose fermentations (IBB10B05). Enzymatic hydrolyzates were prepared from up to 15% dry mass pretreated (steam explosion) wheat straw and contained glucose and xylose in a mass ratio of approximately 2.

**Results:**

With all strains, yield coefficients based on total sugar consumed were high for ethanol (0.39 to 0.40 g/g) and notably low for fermentation by-products (glycerol: ≤0.10 g/g; xylitol: ≤0.08 g/g; acetate: 0.04 g/g). In contrast to the specific glucose utilization rate that was similar for all strains (*q*_Glucose_ ≈ 2.9 g/g_cell dry weight (CDW)_/h), the xylose consumption rate was enhanced by a factor of 11.5 (IBB10A02; *q*_Xylose_ = 0.23 g/g_CDW_/h) and 17.5 (IBB10B05; *q*_Xylose_ = 0.35 g/g_CDW_/h) as compared to the *q*_Xylose_ of the non-evolved strain BP10001. In xylose-supplemented (50 g/L) hydrolyzates prepared from 5% dry mass, strain IBB10B05 displayed a *q*_Xylose_ of 0.71 g/g_CDW_/h and depleted xylose in 2 days with an ethanol yield of 0.30 g/g. Under the conditions used, IBB10B05 was also capable of slow anaerobic growth.

**Conclusions:**

Laboratory evolution of strain BP10001 resulted in effectively enhanced *q*_Xylose_ at almost complete retention of the fermentation capabilities previously acquired by metabolic engineering. Strain IBB10B05 is a sturdy candidate for intensification of lignocellulose-to-bioethanol processes.

## Background

Second-generation biofuel production aims at biotechnological conversion of lignocellulosic biomass into liquid fuels, typically ethanol. Processes currently advancing to commercial scale production are facing two problems in particular. Firstly, for an efficient release of fermentable sugars from the insoluble feedstock, a technically complex and energy-intensive series of upstream processing steps are required [1-3]. Hence, mechanical and thermochemical pretreatment methods, alone or in combination, are applied to degrade and remove the lignin and to enhance the accessibility of the structural carbohydrates hemicellulose and cellulose for the subsequent enzymatic hydrolysis. During this pretreatment, however, secondary decomposition processes lead to formation of by-products, for example furans, phenolic compounds and organic acids, and many of those are inhibitory or even toxic to microorganisms applied to sugar fermentation [1-3]. It is widely accepted therefore that lignocellulose hydrolyzates constitute exceptionally difficult substrates for biotechnological conversions [1,2,4,5]. Since intermediate purification of the hydrolyzate is usually not a viable process option, a key requirement for efficient second-generation bioethanol production is a microbial strain that combines useful fermentation capabilities with high robustness to the overall conditions of the hydrolyzate [1,2,4,5]. *Saccharomyces cerevisiae* is a sturdy ethanol producer with long-standing history in the fermentation industries. Among the different candidate microorganisms considered, therefore, *S. cerevisiae* stands out as a highly promising choice for industrial scale applications [6].

**Figure 1:**
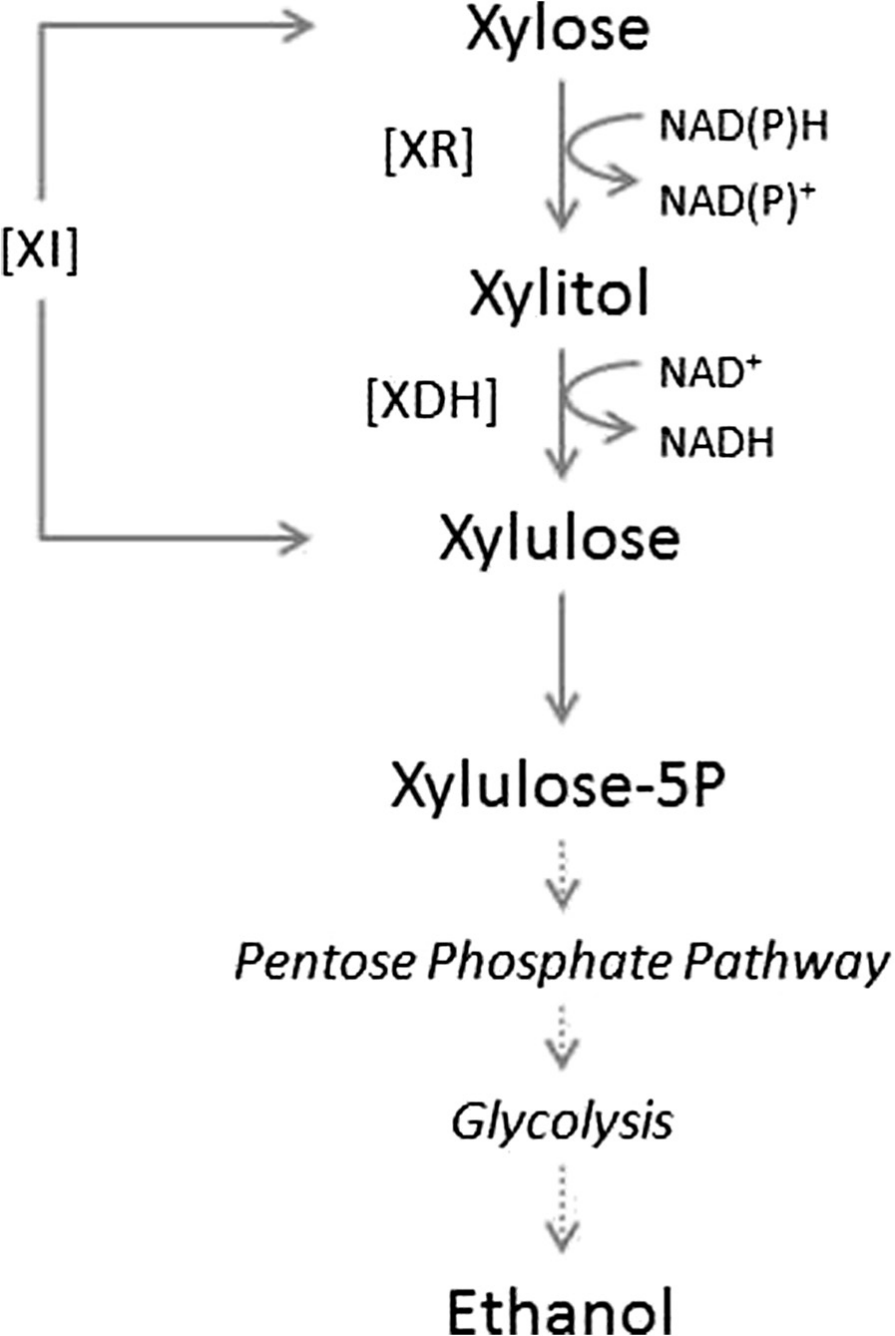
**The two xylose assimilation pathways.** XDH, xylitol dehydrogenase; XI, xylose isomerase; XR, xylose reductase.

Even though the composition of fermentable sugars in lignocellulose hydrolyzates varies strongly depending on the feedstock and the upstream processing technology applied, it is typical for most substrates to contain a significant amount of xylose next to the main glucose [1]. A major limitation of *S. cerevisiae* for lignocellulosic bioethanol development is the organism’s natural inability to utilize xylose. Metabolic engineering has therefore been key in the development of xylose-fermenting strains of *S. cerevisiae *[2,5-8]. The applied strategies can be classified broadly according to whether xylose assimilation, which occurs through net isomerization of xylose into xylulose, was achieved via two-step oxidoreductive or direct isomerase-catalyzed transformation, as shown in Figure 1 [9-13]. Recombinant strains derived from either strategy displayed the expected broadening of substrate scope towards xylose. However, their specific xylose uptake rates and ethanol formation rates were still very low in comparison to the corresponding specific rates on glucose [5-7]. Moreover, xylose fermentation capabilities were usually severely deteriorated upon switching from synthetic substrate conditions to the harsher conditions of a lignocellulosic hydrolyzate [2,6,8,14]. A particular downside of strains harboring the oxidoreductive pathway is that a substantial amount of xylose is converted to xylitol and thus lost for ethanol production. Xylitol by-product formation is widely believed to have its origin in a mismatched coenzyme usage, NADP or NAD, during xylose reduction and xylitol oxidation (Figure 1) [11,15-19]. Coenzyme specificity engineering in xylose reductase (XR; NADPH → NADH) and xylitol dehydrogenase (XDH; NAD^+^ → NADP^+^) was useful to render the two-step isomerization of xylose a more nearly redox-neutral process (Figure 1) [15-17,20]. However, aside from the intended change in coenzyme specificity, engineered enzymes must also fulfill the requirement of good activity under physiological boundary conditions *in vivo*. Due to their favorable kinetic properties that include high turnover number and apparent substrate and coenzyme affinities well aligned to intracellular metabolite concentrations in xylose-fermenting *S. cerevisiae*, some of the reported XR variants are especially promising for strain development. This is exemplified clearly by strain BP10001 from this laboratory that harbors an optimized NADH-preferring mutant of *Candida tenuis* XR. BP10001 shows a xylitol yield (*Y*_Xylitol_) more than halved by comparison to *Y*_Xylitol_ in the reference strain BP000 that expresses the NADPH-preferring wild-type XR [16,18-22]. Importantly, the lowering of *Y*_Xylitol_ in BP10001 was not achieved in a trade-off against a decrease in xylose uptake rate (*q*_Xylose_) [20,22]. However, *q*_Xylose_ of strain BP10001 was still almost two magnitude orders lower than the corresponding *q*_Glucose_ and it was certainly insufficient for viable co-fermentation of glucose and xylose [20,22]. It must be emphasized that low *q*_Xylose_ is a common problem of xylose-fermenting strains of *S. cerevisiae*, regardless of the metabolic engineering strategy applied in their construction [6,7,13].

The specific substrate consumption rate is a kinetically complex rate parameter, which often eludes clear-cut dissection into one or more rate-determining transport or reaction steps, these in turn presenting distinct targets for further metabolic engineering. Laboratory evolution presents a long-known method for physiology optimization in microorganisms, and it has recently been adapted as a powerful complement of metabolic engineering to the development of high *q*_Xylose_ strains of *S. cerevisiae *[23-26]. Improvements in *q*_Xylose_ of up to one magnitude order were achieved using evolutionary engineering, and strains capable of co-utilization of glucose and xylose, and of cellobiose and xylose were obtained [25-28]. Interestingly, some strains also acquired the ability of slow anaerobic growth as a result of the evolution, presumably as a consequence of the enhanced ATP production rate at elevated *q*_Xylose _[25,26]. Moreover, resistance to the overall environment of lignocellulose hydrolyzates or certain compounds present in it (for example furfural, acetic acid) could also be improved substantially by evolutionary engineering [23,29].

We have therefore applied laboratory evolution to strain BP10001 and used specific anaerobic growth rate (μ_Xylose_) in combination with high *q*_Xylose_ to select strain IBB10A02 from several anaerobically growing yeast strains thus obtained. Further strain adaption through multiple rounds of batch xylose fermentations resulted in the identification of strain IBB10B05. The two evolved strains plus their parent strain BP10001 were compared in mixed glucose-xylose fermentation of undiluted and non-detoxified wheat straw hydrolyzate, which represents a notably challenging substrate. In Europe, wheat straw is considered to have the highest potential as biomass source for bioethanol production due to its abundance and low cost [30]. Even though wheat straw hydrolyzates have already been utilized as substrate for bioethanol production in the past, efficient xylose conversion often had complex process requirements, for example simultaneous saccharification and fermentation (SSF) [31,32] or fed-batch processing [33]. Therefore, further improvement in xylose conversion rates and higher ethanol yields at lower by-product formation must still be rendered possible [4-6,29,34]. To ensure optimal conditions for xylose fermentation and to keep technical requirements to a minimum level the process was run as separate hydrolysis and co-fermentation (SHCF) with the fermentation accomplished in simple batch cultures [33,34]. Results presented in this paper delineate marked gain in xylose fermentation efficiency and overall substrate tolerance due to evolutionary engineering, and strain IBB10B05 was identified as a promising candidate for direct glucose-xylose fermentation in unprocessed wheat straw hydrolyzate.

## Results and discussion

### Composition of the feedstock, and preparation of the sugar substrate for fermentation

Steam explosion has been described as an efficient and cost-effective method for the pretreatment of wheat straw [3,31-36]. Auxiliary chemical treatment is often applied to reinforce effectiveness of the steam explosion. We have applied here simple pretreatment based on steam explosion only. Table 1 shows the results of compositional analysis of the wheat straw after pretreatment. Dry matter content was about 20%, the water-insoluble portion thereof being roughly 68%. Pretreated feedstock composition was in agreement with literature data on wheat straw samples from different origin, but processed similarly [32,34,35].

**Table 1:** Compositional analysis of the pretreated wheat straw

**Component in dry matter**	**Percentage (%)**
Carbohydrates	
Glucose	47.8
Xylose	21.3
Others	2.8
Non-carbohydrates	
Acid-soluble lignin	3.8
Acid-insoluble lignin	18.0
Ashes	1.5

Mixed sugar substrates for yeast fermentation were prepared by enzymatic saccharification of the pretreated wheat straw at a dry matter loading of 5% or 15% (by weight). Both fermentations were accomplished at the same boundary conditions, for example pH, temperature, agitation and starting OD_600_. However, medium supplementation and sugar composition (glucose and xylose ratio) varied between the two hydrolyzates. We noticed that regardless of substrate and enzyme loadings applied to the saccharification, the resulting hydrolyzates always contained double the amount of glucose compared to xylose. Thus, 5% hydrolyzates contained glucose and xylose in concentrations of approximately 14 g/L and 7 g/L, respectively. To investigate xylose fermentation at elevated xylose/glucose (Xyl/Glc) ratio, like done in various studies from this and other groups in the past [32,34,35], we supplemented the 5% hydrolyzate to a final xylose concentration of approximately 50 g/L, resulting in a Xyl/Glc ratio of approximately 4. Mineral media, containing salts, vitamins and trace elements, was added to 5% hydrolyzates to ensure optimal fermentation conditions and maximal viability of the yeast [24,32,35]. However, in any larger scale process media additives such as salts or vitamins are economically and procedurally not feasible [2,14,37]. To address this problem, further fermentation studies were conducted under most simplified process and substrate conditions in the highly concentrated, non-detoxified 15% hydrolyzate with yeast extract as sole media supplement. As described previously, yeast extract serves as an excellent complex media additive for the fermentation of wheat straw hydrolyzates with high dry matter loadings [38]. Economically advantageous solutions, such as corn steep liquor or urea, in contrast, were described to be insufficient for wheat straw to bioethanol processes [38]. In this study, yeast extract served as a model for complex media supplementation, replacing the expensive mineral medium. However, addition of cheaper nutrient and nitrogen sources, for example grass juice, are future targets for process optimization.

Utilization of high substrate loadings is beneficial for lignocellulosic bioethanol production since the increase in sugar content (here: approximately 40 g/L and 20 g/L glucose and xylose, respectively in the 15% hydrolyzate) results in higher ethanol titers, which is important for facilitation of downstream processing. Throughout the manuscript, the sugar substrates used are identified as 15% hydrolyzate and 5% hydrolyzate_X_, where subscript ‘_X_’ indicates externally added xylose.

### Effect of *S. cerevisiae* strain evolution on xylose fermentation in basal medium

Strains IBB10A02 and IBB10B05 were obtained by laboratory evolution as described under Methods. The two strains were compared to their progenitor strain BP10001 by evaluating xylose (58 g/L) utilization in anaerobic shaken flask cultures. Time courses of fermentation product formation and biomass growth during xylose conversion were recorded for each strain, and the results are shown in Additional file 1. Compared to BP10001, the evolved strains displayed enhanced xylose fermentation capabilities in several respects. First of all, xylose consumption was markedly accelerated due to the combined effects of a distinct (≥2.5-fold) increase in *q*_Xylose_ and the establishment of anaerobic growth caused by the laboratory evolution (Table 2). *q*_Xylose_ values of about 1.0 g/g_CDW_/h are among the highest reported for xylose-fermenting strains of *S. cerevisiae *[9,11,22,23,26,39]. Strain IBB10B05 grew faster and to a higher biomass concentration than strain IBB10A02 (Table 2, Additional file 1). In both strains, however, the specific growth rate on xylose (μ_Xylose_) decreased strongly as xylose conversion progressed. Growth ceased completely at extended fermentation time (≥120 h), even though more than half of the initial xylose was still present and utilization of the sugar substrate continued further on. Considering that *q*_Xylose_ also decreased appreciably over the fermentation time course, shutdown of growth may reflect a drop of *q*_Xylose_ (and the ATP production rate associated with it) below a critical value. Additionally to its effects on key rate parameters of the fermentation (Table 2), we further analyzed the effect of laboratory evolution on the product distribution pattern of external metabolites produced from xylose. Data are summarized in Additional file 2. For all three yeast strains, the ethanol yield coefficient (*Y*_Ethanol/Xylose_) was approximately 0.31 g/g. Yield coefficients for glycerol (*Y*_Glycerol/Xylose_) were also similar for strains BP10001, IBB10A02 and IBB10B05 at approximately 0.04 g/g. Observed xylitol yields (*Y*_Xylitol/Xylose_) were comparable for strain BP10001 and IBB10A02 (0.15 g/g) and increased 1.3-fold in fermentation utilizing strain IBB10B05 (0.19 g/g). The yield coefficients for strain BP10001 agree well with previously published results [22], indicating that the switch from mineral to yeast extract medium had no influence on product formation. Accordingly, mixed glucose-xylose conversion in spent sulfite liquor utilizing IBB10B05 was not affected by replacing mineral media with yeast extract [40]. Results of fermentation of xylose as the sole sugar substrate (Additional files 1 and 2, Table 2) strongly supported application of the two evolved yeast strains for mixed glucose-xylose conversion in wheat straw hydrolyzates. Conditions in the lignocellulose hydrolyzate are however noteworthy different from those of a pure glucose-xylose substrate (see, for example Palmqvist and Hahn-Hägerdal [4], Hahn-Hägerdal *et al*. [6], Casey *et al*. [41]), and the switch from defined to technological sugar substrates has proven to be difficult in the past [2,4,6].

**Table 2:** **Comparison of μ**_**max **_**and *q***_**X** **ylose **_**of strains BP10001, IBB10A02 and IBB10B05 obtained from xylose fermentation in YX medium**

**Parameter**	**BP10001**	**IBB10A02**	**IBB10B05**
*q*_Xylose_ (g/g_CDW_/h)	0.37 ± 0.05	0.98 ± 0.08	1.04 ± 0.06
μ_max_ (h^-1^)	n.d.	0.017 ± 0.001	0.021 ± 0.001

Data was obtained from two independent fermentations. n.d., not detectable.

### Mixed glucose-xylose fermentation in xylose enriched 5% hydrolyzate_X_: laboratory evolution results in markedly accelerated xylose utilization

**Figure 2:**
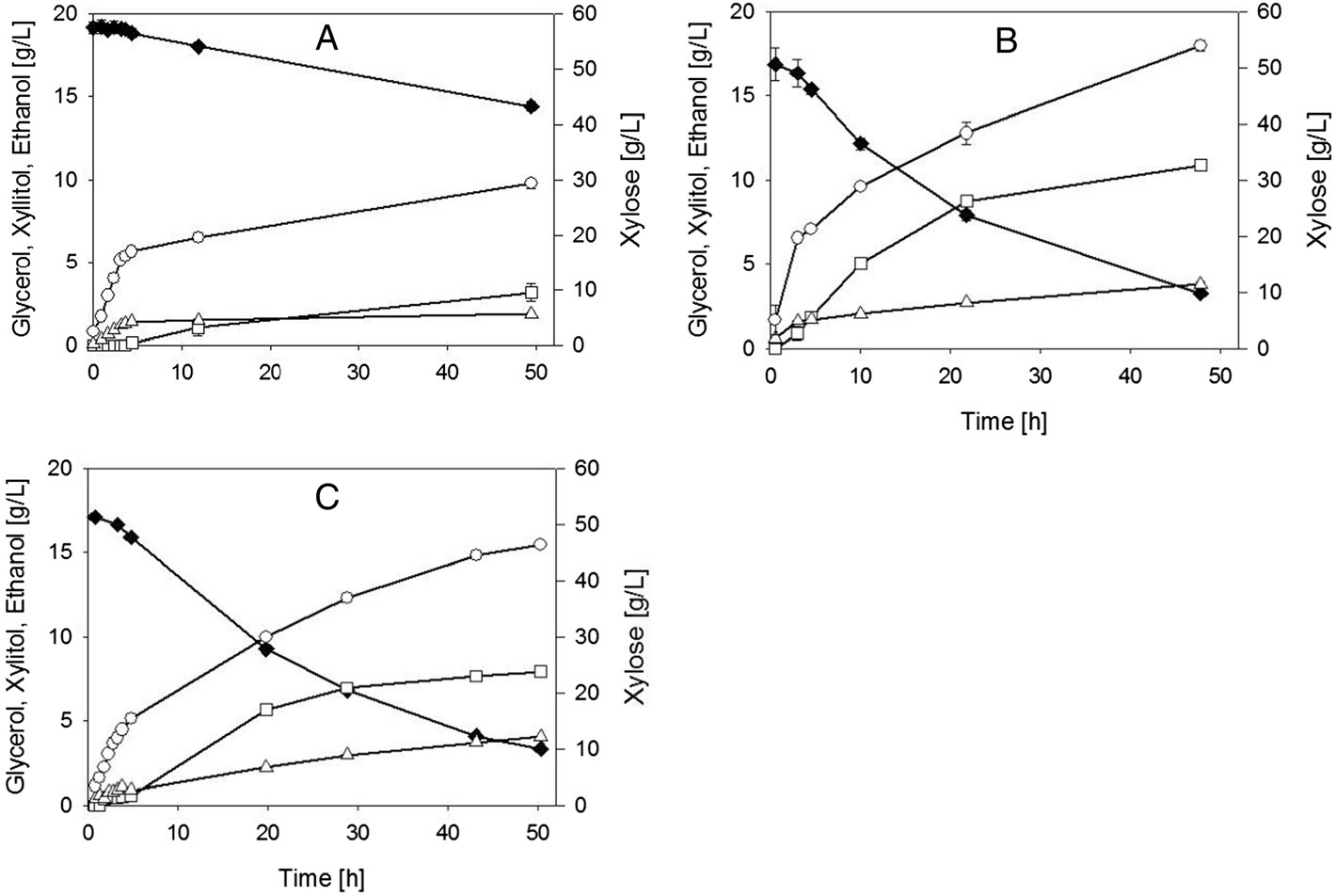
**Time courses of mixed glucose-xylose fermentation in 5% hydrolyzate**_**X**_**.** Depicted are the first 50 h of fermentation using strains **(A)** BP10001, **(B)** IBB10A02 and **(C)** IBB10B05. Glucose (approximately 14 g/L) was depleted within the first 5 h and the ‘glucose phase’ is shown in Additional file 3. Data points are mean values of two independent fermentation experiments. Full diamonds, xylose; empty triangles, glycerol; empty squares, xylitol; empty circles, ethanol.

A detailed time-course analysis for glucose-xylose fermentation in 5% hydrolyzate_X_ was performed, comparing the two evolved *S. cerevisiae* strains to the BP10001 reference. Results are summarized in Figure 2. All strains utilized glucose much faster than xylose. For clarity reasons, therefore, the respective ‘glucose phase’ was singled out and is shown in a separate graph (Additional file 3) depicting only the first phase (approximately 8 h) of the fermentation course. Determination of a specific glucose utilization rate (*q*_Glucose_) for each strain was hampered due to rapid substrate depletion. However, time resolution of the shown data (Additional file 3) was sufficient to clarify that the three yeast strains consumed glucose at a comparable rate. Ethanol production (*Y*_Ethanol_ = approximately 0.40 g/g) and glycerol formation (*Y*_Glycerol_ = approximately 0.06 g/g) in the glucose phase were also similar for the different strains (Additional file 3). The specific growth rate (μ_Glucose_) for strain IBB10B05 approached a value expected for uninhibited *S. cerevisiae* growth during glucose fermentation, while in strains IBB10A02 and BP10001, the μ_Glucose_ values were notably decreased (Table 3). This provided the first evidence that strain IBB10B05 had gained superior resistivity to the conditions of the wheat straw hydrolyzate. Overall, IBB10B05 grew to a biomass concentration of approximately 2.6 g/L, which is significantly higher than reported for other yeast strains under comparable substrate conditions [34].

**Table 3:** **Physiological parameters of strains BP10001, IBB10A02 and IBB10B05 obtained from mixed glucose-xylose fermentation in 5% hydrolyzate**_**X**_

**Parameter**	**BP10001**	**IBB10A02**	**IBB10B05**
*q*_Glucose_ (g/g_CDW_/h)	n.d.	n.d.	n.d.
*q*_Xylose_ (g/g_CDW_/h)	0.15 ± 0.01	0.53 ± 0.05	0.71 ± 0.01
μ_max_^a^	0.09 ± 0.01	0.13 ± 0.02	0.43 ± 0.03
*Y*_Ethanol_ (g/g)	0.35	0.31	0.30
(*Y*_Ethanol/available sugars_ (g/g))^b^	(0.18)	(0.25)	(0.29)
*Y*_Glycerol_ (g/g)	0.06	0.06	0.09
*Y*_Xylitol_ (g/g)	0.15	0.22	0.17
*Y*_Acetate_ (g/g)	0.04	0.06	0.05
*Y*_BM_ (g/g)	0.02	0.03	0.04
C-recovery (%)	98 ± 1	102 ± 1	96 ± 1

Data was obtained from two independent fermentations, mean errors of product coefficients were always below 11%. Fermentation time courses are shown in Figure 2. Fermentation in 5% hydrolyzate_X_: glucose to xylose ratio of approximately 0.2.^a^ Determined in the first 4.5 hours of fermentation;^b^*Y*_Ethanol/available sugars_ = c (Ethanol produced in 100 h of fermentation)/c (Available glucose and xylose). Note that utilization of xylose was much lower in strain BP10001 than it was in the evolved strains. This affects the calculated ethanol yield coefficient based on total sugar (glucose and xylose) consumed. The ethanol yield coefficient was therefore also expressed based on total sugars available in the reaction, as shown in parenthesis. n.d., not detectable.

Evaluation of the second phase of the fermentation time courses, where xylose was utilized (Figure 2), revealed significant differences between the two evolved strains and their progenitor strain. The corresponding rate parameters and yield coefficients are summarized in Table 3. *q*_Xylose_ of IBB10A02 was enhanced 3.5-fold as compared to BP10001. IBB10B05 even surpassed IBB10A02 in terms of *q*_Xylose_. The switch from pure xylose substrate to 5% hydrolyzate_X_ caused a 2.5-fold decrease in *q*_Xylose_ for BP10001. In contrast, the evolved strains, particularly IBB10B05, showed a much less pronounced drop in *q*_Xylose_ (Table 3). It is noteworthy that both evolved strains consumed nearly all of the offered 50 g/L xylose within approximately 2 days. BP10001 by contrast showed much smaller xylose utilization (≤10 g/L) in the same time frame (Figure 2). Yield coefficients were similar for each strain, as shown in Table 3 where yield coefficients were calculated on the basis of total sugar, glucose and xylose consumed. Considering bias in the calculated yield coefficients due to unequal xylose utilization by BP10001 as compared to the evolved strains, we compiled a second set of yield coefficients (Additional file 4), which were determined from the xylose phase only, starting when the glucose was depleted fully. *Y*_Ethanol/Xylose_ was about 0.31 ± 0.01 g/g in all strains and xylitol was the main by-product with *Y*_Xylitol/Xylose_ in the range 0.18 to 0.23 g/g. Even though loss of xylose into xylitol formation was substantial with all three strains examined and therefore presents a clear target for strain optimization in the future, it is nevertheless worth noting that *Y*_Xylitol/Xylose_ was not affected by change in substrate from pure xylose to 5% hydrolyzate_X_. It was previously shown that enhanced burden on yeast physiology under conditions of a technological substrate could not only affect *q*_Xylose_, but also result in increased xylitol yields at the expense of ethanol production [40]. In fact, this effect was also observed when substrate was altered from 5% hydrolyzate_X_ to 15% hydrolyzate, as we will show hereinafter. Ethanol formation was almost doubled in the evolved strains compared to BP10001, whereby strain IBB10A02 accumulated up to 18 g/L ethanol within 2 days (Figure 2). The slightly smaller volumetric ethanol production by strain IBB10B05 compared to strain IBB10A02 is ascribed to experimental differences in the biomass concentration at the time of inoculation. Based on total sugar consumed, the obtained ethanol yield was about 70% of the theoretical value. Contrary to fermentations carried out with pure sugar substrate (Table 2), none of the three strains showed anaerobic growth on xylose in the 5% hydrolyzate_X_. Although cell growth is an important feature of *S. cerevisiae* strains applied to lignocellulose-to-bioethanol processes [6,7,26], growth rates (μ_Glucose_ and μ_Xylose_) are often excluded in literature [31-33,35], leading to the assumption that those yeast strains might have been growth impaired [34].

### Mixed glucose-xylose fermentation in undiluted and non-detoxified 15% hydrolyzate: laboratory evolution confers a high degree of strain robustness

**Figure 3:**
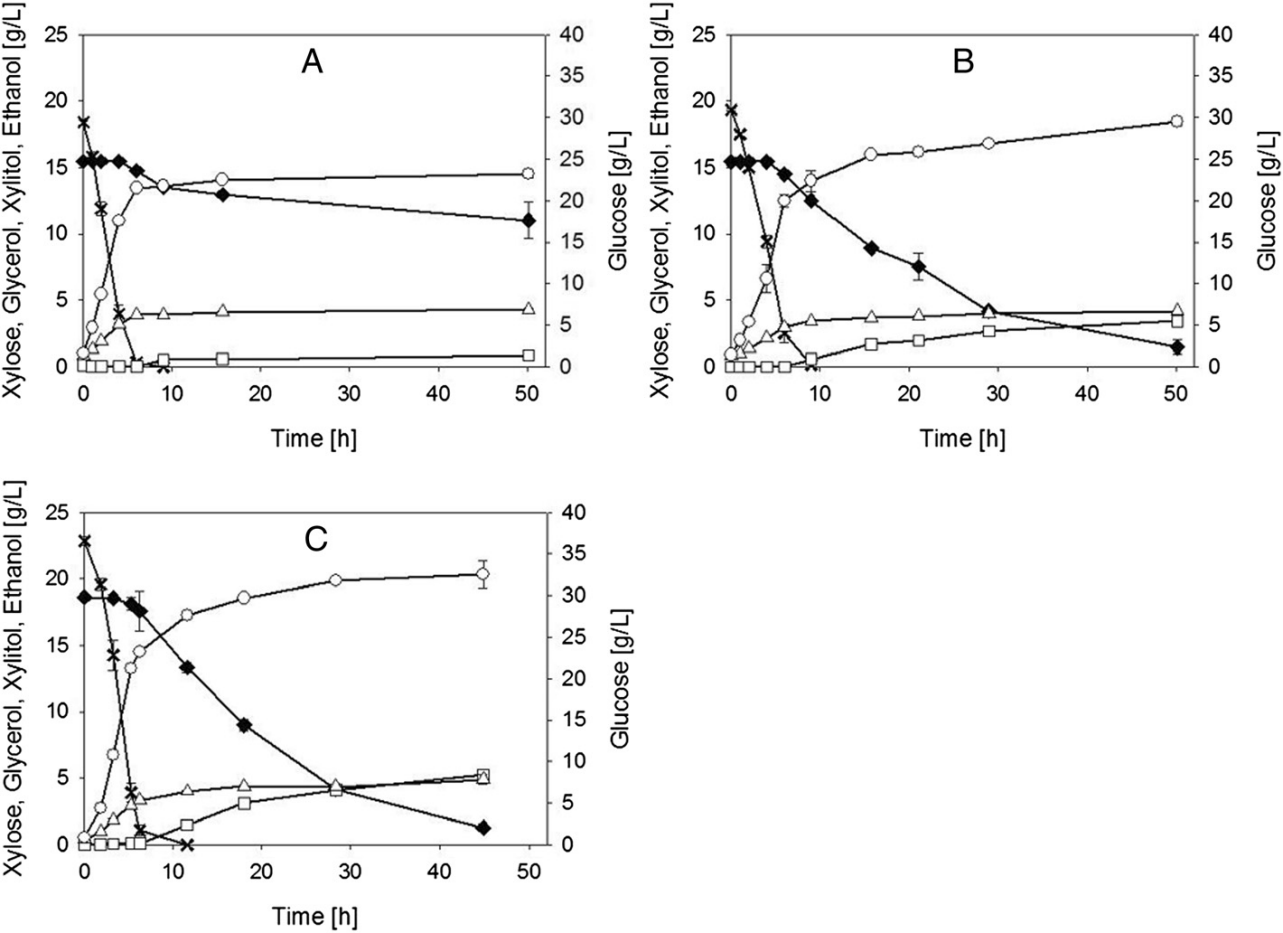
**Time courses of mixed glucose-xylose fermentation in 15% hydrolyzate.** Depicted are the first 50 h of fermentation utilizing strains **(A)** BP10001, **(B)** IBB10A02 and **(C)** IBB10B05. Data points are mean values of two independent fermentation experiments. Crosses, glucose; full diamonds, xylose; empty triangles, glycerol; empty squares, xylitol; empty circles, ethanol.

Results showing that laboratory evolution had caused enhancement of *q*_Xylose_ and consolidated anaerobic growth (on glucose) without compromising ethanol yield during mixed glucose-xylose fermentation of the 5% hydrolyzate_X_ prompted us to take conversion experiments to another level of substrate complexity. Impairment of xylose fermentation in recombinant *S. cerevisiae* was previously described, when applying undiluted substrate at similar concentration as presented in this study [24,34,35], and this will also be confirmed for strain BP10001 hereinafter. Fermentation time courses recorded with the two evolved strains and strain BP10001 are shown in Figure 3. Specific rate and yield parameters calculated from the data are summarized in Table 4.

**Table 4:** Physiological parameters of strains BP10001, IBB10A02 and IBB10B05 obtained from mixed glucose-xylose fermentation in 15% hydrolyzate

**Parameter**	**BP10001**	**IBB10A02**	**IBB10B05**
*q*_Glucose_ (g/g_CDW_/h)^a1^	2.81 ± 0.04	1.71 ± 0.05	2.90 ± 0.22
*q*_*X*ylose_ (g/g_CDW_/h)	0.02 ± 0.01	0.23 ± 0.01	0.35 ± 0.02
μ_max_^a2^	0.10 ± 0.01	0.10 ± 0.01	0.19 ± 0.01
*Y*_Ethanol_ (g/g)	0.40	0.39	0.39
(*Y*_Ethanol/available sugars_ (g/g))^b^	(0.13)	(0.38)	(0.38)
*Y*_Glycerol_ (g/g)	0.10	0.08	0.08
*Y*_Xylitol_ (g/g)	0.04	0.08	0.08
*Y*_Acetate_ (g/g)	0.04	0.04	0.04
*Y*_BM_ (g/g)	0.05	0.04	0.06
C-recovery (%)	103 ± 1	101 ± 1	104 ± 1

Data was obtained from two independent fermentations, mean errors of product coefficients were always below 14%. Fermentation time courses are shown in Figure 3. Fermentation in 15% hydrolyzate: glucose to xylose ratio of approximately 2.^a^ Determined in the first ^a1^ 3.5 h and ^a2^ 6 hours of fermentation;^b^*Y*_Ethanol/available sugars_ = c (Ethanol produced in 50 h of fermentation)/c (Available glucose and xylose).

A recurring pattern in the fermentation time courses in Figure 3 was their division into two phases according to sugar substrate utilization. Glucose was consumed much faster than xylose. At the resolution of the experimental data with respect to time and concentration, sugar consumption appeared to have been largely sequential, glucose prior to xylose. It was shown in prior studies of BP10001 and also other xylose-fermenting strains of *S. cerevisiae* that low concentrations of glucose stimulate the uptake of xylose and only under these conditions a significant amount of true co-utilization of glucose and xylose becomes eventually possible [22,42]. However, at glucose concentrations at or higher than 5 g/L, xylose consumption is inhibited [22,42]. The evolved strains IBB10A02 and IBB10B05 do in fact show a small amount of glucose-xylose co-utilization at the end of their respective glucose phase (Figure 3). Strain BP10001 utilizes xylose at a much slower rate by comparison, thus resulting in a completely sequential fermentation pattern.

Recently *S. cerevisiae* harboring xylose isomerase (Figure 1) was evolved to a *q*_Xylose_ exceeding that of IBB10B05 in pure xylose substrate by still a factor of about 2 [39]. Glucose-xylose fermentation by the resulting yeast strain occurred at the transition between sequential and simultaneous utilization of hexose and pentose substrates, indicating that true co-fermentation may become possible at sufficiently high *q*_Xylose_. However, fermentation of lignocellulose hydrolyzates was not examined, and evidence from this study suggests that *q*_Xylose_ is more strongly affected by substrate conditions than *q*_Glucose_. Moreover, a number of publications on mixed glucose-xylose fermentation in lignocellulose hydrolyzates by recombinant *S. cerevisiae*, typically strains constructed using the XR/XDH pathway, agree with our findings of predominantly sequential sugar substrate utilization [33-35].

Even though fermentation of glucose was fast in each case (Figure 3), the three yeast strains differed in respect to *q*_Glucose_ and μ_Glucose_, thus resulting in distinctly different efficiencies of glucose conversion. Strain IBB10B05 showed the highest μ_Glucose_ and the fastest glucose utilization (Figure 3, Table 4). *q*_Glucose_ of strain IBB10A02 was surprisingly low. Analysis of the xylose phase from the 15% hydrolyzate conversion time course revealed dramatic effects of laboratory evolution on the yeast strains’ xylose fermentation capabilities. While the progenitor strain BP10001 was utterly inefficient in utilizing xylose, the two evolved strains converted (nearly) all of the xylose present in 15% hydrolyzate within about two days. Expressed in *q*_Xylose_, laboratory evolution brought about 11.5-fold (IBB10A02) and 17.5-fold (IBB10B05) enhancement of xylose utilization in 15% hydrolyzate as compared to BP10001. These improvement factors are of remarkable magnitude, and they therefore underscore the huge potential of evolutionary yeast strain engineering for biofuel process development.

Yield coefficients for mixed glucose-xylose fermentation in 15% hydrolyzate indicate good ethanol production (*Y*_Ethanol_ approximately 0.40 g/g total sugars). Xylitol and glycerol were major by-products. A compilation of yield coefficients derived from the xylose phase is provided in Additional file 4. Compared to fermentations conducted in YX and 5% hydrolyzate_X_ media, xylitol yields (*Y*_Xylitol/Xylose_) in fermentations of the 15% hydrolyzate were notably elevated (up to 0.30 g/g; Additional files 2 and 4). The reason for high xylitol formation in 15% hydrolyzate was not further pursued.

Although direct comparison is difficult due to different feedstock applied, we noticed that *Y*_Ethanol_ (approximately 0.40 g/g total sugars) and the final ethanol titer (approximately 20 g/L) for 15% hydrolyzate conversion by the evolved strains IBB10A02 and IBB10B05 (Table 4, Figure 3) were superior to the same fermentation parameters reported from other studies, where *Y*_Ethanol_ did not exceed values of typically 0.37 g/g total sugars when undiluted substrate was applied, and final ethanol titers were below 10 g/L [34,35]. Higher ethanol yields as well as enhanced final ethanol titers were only achieved when glucose was fed continuously to the fermentation mixture, by running the process in SSF [32,43] or separate hydrolysis and fermentation (SHF) run in fed-batch mode [33,34]. Consistently, we have shown that *q*_Xylose_ in mixed glucose-xylose fermentations by strain BP10001 is accelerated substantially by feeding low levels of glucose [22]. However, requirement for controlling the glucose feed tightly adds complexity of the process operation. Yeast strains producing high amounts of ethanol from xylose in simple batch fermentations are therefore of considerable interest for application in large-scale bioprocessing.

### Strain evolution as tool for process intensification: comparison of yeast strain performance under different substrate conditions

Evidence from different studies, including the work presented herein, strongly supports the suggestion that evolutionary engineering of *S. cerevisiae* constitutes a powerful approach to achieve significant process intensification for xylose-to-ethanol fermentation [23-26,34,39]. Improvements in specific rate parameters and yield coefficients were shown to translate directly into pronounced enhancement of the final ethanol concentration, the process productivity, or both. Table 5 lists xylose-fermenting yeast strains generated by laboratory evolution and compares each strain to its corresponding progenitor. Consequences of evolutionary engineering were assessed in pure sugar substrate fermentations [23,26,39], but also in lignocellulose hydrolyzate conversions [24,34]. Results show that in a wide range of media and cultivation conditions using pure sugar substrates, evolution caused effective enhancement in *q*_Xylose_ (up to 8-fold) and conferred, or resulted in stabilization of, anaerobic growth on xylose (Table 5). The degree of xylose utilization and thus, the end concentration of ethanol were also increased by up to 4-fold. Even though the different studies are difficult to compare due to large variations in the experimental settings used, it is nonetheless clear that strain IBB10B05 features a notable overall improvement in xylose fermentation capability. Effectiveness of the evolutionary procedure in IBB10B05 was remarkable in particular, considering that development of the progenitor strain BP10001 had involved only a minimum amount of metabolic engineering of the parent strain *S. cerevisiae* CEN.PK 113-5D. Interestingly, change of the sugar substrate to an unprocessed concentrated wheat straw hydrolyzate resulted in substantial (≥2-fold) reinforcement of the process intensification effect of the strain evolution. This is reflected in very pronounced enhancement of *q*_Xylose_, the completeness of xylose consumption, and the final ethanol titer achievable with strain IBB10B05 as compared to strain BP10001. This result serves to emphasize the high robustness acquired by strain IBB10B05 during evolutionary engineering, despite the fact that increased resistance to conditions of the hydrolyzate was not selected for. Remarkably enough, yeast strain evolutions reported from other laboratories to specifically address tolerance against biomass-derived inhibitors did not achieve comparable improvements of strain performance during fermentation of lignocellulose hydrolyzates [24,34]. The lignocellulosic ethanol concentration of 21 g/L reached with strain IBB10B05 therefore surpassed comparative values in Table 5 by 3-fold or more.

**Table 5:** **Laboratory evolution of xylose-fermenting strains of *S. cerevisiae *** **as tool for process intensification: comparison of key process parameters reported for the progenitor strain and the evolved strain, respectively**

**Progenitor strain - evolved strain**	**Fermentation condition**^**b**^	**c**_**Sugar **_**(g/L)**^**c**^	***q***_**Xylose **_**(g/g**_**CDW**_**/h)**	**c**_**Ethanol **_**(g/L)**^**d**^	***Y***_**Ethanol **_**(g/g**_**total sugars**_**)**	**μ**_**max **_**(h**^**-1**^**)**^**e**^	**Source**
***Genetic background***^***a***^							
TMB3001 -	MM	Glc: 50	0.08*	22*	0.38*	0.44^Glc^	[26]
TMB3001C1	AN	Xyl: 50	0.31*	28*	0.40*	0.44^Glc^	
*XR/XDH/XK*	LCD		(3.9–fold)	(1.3–fold)	(1.1–fold)	(–)	
H131-A3^SB-2^ -	YE	Xyl: 40	0.26	4*	0.42	0.06^Xyl^	[39]
H131-A3^CS^	AN		0.94	15*	0.43	0.12^Xyl^	
*XI/ PPP/ T*	LCD		(3.6–fold)	(3.8–fold)	(–)	(2–fold)	
HDY.GUF5 -	YE, Pep	Glc: 36	0.13	18*	0.23	n.a.	[23]
GS1.11-26	Semi-AN	Xyl: 37	1.10	34*	0.46	n.a.	
*XI/PPP*	HCD		(8.5–fold)	(1.9–fold)	(1.8–fold)	(–)	
BP10001 -	YE	Xyl: 50	0.37	0.9	0.30	-	This study
IBB10B05	AN		1.04	2.8	0.31	0.02^Xyl^	
*XR/XDH/XK*	LCD		(2.8–fold)	(3.1–fold)	(–)	(–)	
TMB3400 -	Wheat straw hydrolyzate, YE, salts, pH 5	Glc: 7.6	0.20*	5.5	0.20	-	[34]
KE6-13i	AN	Xyl: 38	0.04*	6	0.27	-	
*XR/XDH/XK*	HCD		(-)	(1.1–fold)	(1.4–fold)	(–)	
TMB3400 -	Spruce hydrolyzate, MM, pH 5		n.a.	7.8*	0.40	0.07^Glc^	[24]
KE1-17	AN	Glc: 18	n.a.	7.9*	0.43	0.08^Glc^	
*XR/XDH/XK*	LCD	Xyl: 9	(–)	(–)	(1.1–fold)	(1.14–fold)	
BP10001 -	Wheat straw hydrolyzate, YE, pH 6.5		0.02	4	0.40	0.10^Glc^	This study
IBB10B05	AN	Glc: 32	0.35	21	0.39	0.19^Glc^	
*XR/XDH/XK*	HCD	Xyl: 16	(17.5–fold)	(5.3–fold)	(–)	(1.9–fold)	

^a^Strain background: PPP, overexpression of genes from the pentose phosphate pathway; T, overexpression of the HXT7 transporter; XDH, xylitol dehydrogenase; XI, xylose isomerase; XK, xylulose kinase; XR, xylose reductase. ^b^AN, anaerobic; HCD, high cell density (start OD_600_ of fermentation ≥1); LCD, low cell density (start OD_600_ of fermentation ≤0.5); MM, mineral medium; Pep, peptone; YE, yeast extract. ^c^Initial sugar concentration of the substrate. ^d^Ethanol produced within the first 50 h of fermentation or earlier, when sugars were depleted before. ^e^Maximal growth rate on glucose (Glc) or xylose (Xyl). n.a., not analyzed; in parenthesis, improvement calculated from the evolved strain as compared to the progenitor strain; *Data are derived from the time courses given in the respective publications.

## Conclusions

The *q*_Xylose_ is a complex physiological parameter of key technological importance in *S. cerevisiae* fermentations of lignocellulose hydrolyzates. Laboratory evolution of strain BP10001 to generate strains IBB10A02 and IBB10B05 resulted in effectively (up to 17.5-fold) enhanced *q*_Xylose_ at complete perpetuation of the fermentation capabilities (*Y*_Ethanol_; *q*_Glucose_) previously acquired by metabolic engineering. Strain IBB10B05 was identified as a particularly robust candidate for intensification of lignocellulose-to-bioethanol production processes.

## Methods

### Chemicals and media used

Unless mentioned otherwise, all chemicals were from Carl Roth + Co KG (Karlsruhe, Germany). Defined mineral (M-) medium was prepared as described elsewhere [15], except that riboflavin and folic acid were not added. For use of M-medium under anaerobic conditions, ergosterol (10 mg/L), Tween 80 (0.42 g/L) and 250 μL/L Antifoam 204 (all from Sigma-Aldrich, St Louis, MO, USA) were additionally supplied. YPD medium contained yeast extract (10 g/L), peptone (from casein, 20 g/L) and glucose (20 g/L). YX medium contained yeast extract (10 g/L) and xylose (58 g/L). Medium for anaerobic agar plate cultivation contained yeast extract (8 g/L), peptone (from casein, 10 g/L), xylose (20 g/L), agar (13 g/L), thioglycolate (500 mg/L), L-cysteine (500 mg/L) and resazurin (1 mg/L). All media were brought to pH 6.5, and the pH was verified after sterilization.

### Laboratory evolution of strain BP10001 and isolation of strains IBB10A02 and IBB10B05

Strain BP10001 was previously constructed from *S. cerevisiae* CEN.PK 113-5D through genomic integration of genes encoding a doubly mutated (Lys^274^-to-Arg; Asn^276^-to-Asp) variant of XR from *C. tenuis* and the wild-type XDH from *Galactocandida mastotermitis*. Another gene copy of the endogenous xylulose kinase 1 was also integrated. Each gene was expressed under control of the constitutive TDH promoter and the CYC1 terminator. Laboratory evolution was carried out with strain BP10001 in two steps. Because evolutionary engineering of BP10001 will be described in a separate paper, only a brief summary is given here. Firstly, strain BP10001 was incubated in 15 mL glass tubes (Pyrex® Brand 9825) containing 10 mL M-medium supplemented with 50 g/L xylose (XM). Each tube was inoculated to a cell density of 0.04 g_CDW_/L and incubation was at 150 rpm in a CERTOMAT BS-1 incubator shaker (Sartorius AG, Göttingen, Germany) at 30°C for 91 days. Afterwards, 400 μL of cell suspension were plated on agar and incubated in an anaerobic jar at 30°C for 15 days. Single colonies were picked, transferred to new agar plates and further incubated for 5 days. Fast growing colonies were selected for cultivation in tubes as described above. The strain showing the highest μ_max_ (strain IBB10A02), determined as the increase in optical density at 600 nm (OD_600_) over time, was used for further evolutionary engineering by repetitive batches. Hence, strain IBB10A02 was grown (start OD_600_ approximately 0.05) under anaerobic conditions in sealed flasks containing XM-medium. At mid-exponential phase (OD_600_ approximately 1), cells were transferred to a new batch (OD_600_ approximately 0.05) containing fresh XM-medium. Cells were again cultivated until the mid-exponential phase was reached. This procedure was repeated until the observed μ_max_ was approximately doubled. Positive strains were isolated under anaerobic conditions and tested with respect to μ_max_ and *Y*_Xylitol_ and the best performing strain was termed IBB10B05.

### Preparation of the lignocellulosic feedstock

Austrian wheat straw was utilized. The wheat straw was air-dried to a water content of approximately 10% (w/w) and the fibers were chaffed in a shredder (GE 365; Viking, Tyrol, Austria) to reduce the fiber length to an average of 3 to 4 cm. Further, the wheat straw was treated by steam explosion at 200°C, 15 bar for 10 min with a water to wheat straw ratio of 3. After cooling the wheat straw was stored at -20°C in plastic bags. Dry mass (DM) and water-insoluble content (WIS) were analyzed in triplicates. For DM determination, a moisture analyzer operated at 105°C (MA 50; Sartorius AG) was used. For WIS determination, 2 g of the wheat straw was washed with 50 mL of 50°C warm water, dried at 105°C for 24 h and weighed. Additionally, the content of structural carbohydrates, lignin and ash in the wheat straw was analyzed in double determination, following the protocol of the National Renewable Energy Laboratory (NREL) [44]. The resulting compositional analysis is depicted in Table 1. Besides the main sugars glucose and xylose, only small amounts of mannose (<1.9% DM wheat straw) and arabinose (<0.9% DM wheat straw) could be detected, and they are summarized as ‘others’ in Table 1.

### Enzymatic hydrolysis

Enzymes for wheat straw hydrolysis were produced using the *Trichoderma reesei* strain SVG17 as described previously [45]. Briefly, the fungus was cultivated in a BIOSTAT C4 bioreactor (Sartorius AG) with 5 L working volume. Pretreated wheat straw (3% (w/v)) was the sole carbon source. Fermentations were run for 7 to 9 days (30°C, pH 4.5, 20% dissolved oxygen), until no further increase in cellulase activity was detected. Cellulase activity was measured with the filter paper unit (FPU) assay as recommended by the International Union of Pure and Applied Chemistry (IUPAC) [46]. The enzyme solution was harvested by centrifugation (4,420 *g*, 4°C, 20 min, Sorvall RC-5B; DuPont Instruments, Wilmington, DE, USA) and the supernatant filtered sterile (Whatman Klari-Flex System; GE Healthcare, Little Chalfont, UK). Hydrolyzates were freshly prepared shortly before fermentation from one batch of pre-treated wheat straw. The substrate loading was 5% and 15% DM wheat straw and the enzyme loading was 25 FPU/g DM. Reaction was performed in 10 mM sodium acetate buffer (pH 4.8) in 500 mL shaken flasks with ground in stoppers filled with 200 mL wheat straw suspension. The wheat straw suspension was autoclaved and the enzyme solution sterile filtrated. Incubation was at 50°C and 200 rpm in an incubator shaker (CERTOMAT BS-1) for 48 h. Afterwards, the hydrolyzate was heated to 100°C for 15 min and remaining solids were removed by centrifugation (4,420 *g*, 4°C, 10 min, Sorvall RC-5B). The pH of the hydrolyzate was set to 6.5 with 1 M NaOH solution. The sugar content of the hydrolyzates was analyzed by HPLC as described below. We noted variation in the composition of the 15% hydrolyzates prepared in different hydrolysis runs (N >10). Glucose and xylose were present at 42.8 ± 3.9 g/L and 21.1 ± 3.1 g/L, respectively. Acetic acid concentration was 3.6 ± 0.5 g/L. Mannose (<0.7 g/L) and galactose (<0.2 g/L) were present in small amounts. Cellobiose showed the highest variation in the range 1 to 5 g/L. Activity of β-glucosidase, which is the enzyme hydrolyzing cellobiose into glucose, may have been limiting in some of the cellulase preparations applied to hydrolysis. Reasons for variation in sugar content of different hydrolyzates are not completely clear at this time, and their examination was left for consideration in the future. However, each yeast strain was used in multiple fermentation experiments (N ≥3) and the reported parameters were not affected significantly by the relevant variations in hydrolyzate composition.

### Shaken bottle fermentations

Reactions were performed anaerobically at 30°C. About 80% of the total volume was wheat straw hydrolyzate and the remainder volume was composed of media supplementation (10%) and inoculum (10%). In fermentations of 5% hydrolyzate, M-medium and xylose (58 g/L) were added (5% hydrolyzate_X_). The 15% hydrolyzate fermentations were supplemented with yeast extract (10 g/L). Starting OD_600_ in fermentations of the hydrolyzates was 5. Additionally, fermentations were conducted in YX media, with a starting OD_600_ of 0.5. Seed and starter cultures were prepared in M-media with additional glucose (20 g/L) for fermentations supplemented with mineral media. All others were prepared in YPD media. Yeast strains were stored at -70°C in glycerol stocks and initially plated on YPD agar. Incubation was at 30°C for 48 h. Afterwards, cells were transferred to 500 mL shaken flasks filled with 50 mL of the respective media and incubated at 30°C overnight. Subsequently, cells were transferred to 300 mL of fresh media in 1,000 mL shaken flasks. Starting OD_600_ was 0.05 and incubation was at 30°C until the exponential growth phase was reached. Cells were harvested by centrifugation (4,420 *g*, 4°C, 20 min, Sorvall RC-5B) and the cell pellet was washed and resuspended in NaCl solution (9 g/L). Fermentations were accomplished in glass bottles tightly sealed with rubber septa (90 mL working volume). The bottles were sparged with N_2_ prior to and shortly after inoculation. Incubation was at 30°C and 180 rpm (CERTOMAT BS-1).

### Sampling and quantitative analysis of sugars and metabolites

Samples of 1.5 mL were frequently removed from yeast fermentations, centrifuged (15,700 *g*, 4°C, 10 min, Centrifuge 5415 R; Eppendorf, Hamburg, Germany) and the supernatant stored at -20°C for HPLC analysis. Cell growth was recorded as increase in OD_600_. Cell dry weight (CDW) was determined as follows. 10 mL of cell suspension was harvested by centrifugation (3,220 *g*, 4°C, 10 min, Centrifuge 5810 R; Eppendorf), and the cell pellet washed with 10 mL and resuspended in 1 mL NaCl solution (9 g/L). Subsequently, the cell suspension was transferred to pre-dried (105°C, 12 h) glass vials and then dried until weight constancy (105°C, approximately 12 h). The CDW/OD_600_ correlation was determined to be 0.37 and it was established in triple determination. External fermentation products (ethanol, glycerol, acetate and xylitol) were analyzed by HPLC (Merck-Hitachi LaChrom system, L-7250 autosampler, L-7490 RI detector, L-7400 UV detector; Merck, Whitehouse Station, NJ, USA). The system was equipped with an Aminex HPX-87H column and an Aminex Cation H guard column (both Bio-Rad, Hercules, CA, USA). The operation temperature was 65°C and the flow rate of the mobile phase (5 mM sulfuric acid) was 0.6 mL/h. Carbohydrates (glucose, xylose, arabinose, mannose, galactose and cellobiose) were determined with the same HPLC system but equipped with an Aminex HPX-87P column and a de-ashing guard column (both Bio-Rad). Operation temperature was 80°C for the main column and room temperature for the guard column. The mobile phase was deionized water with a flow rate of 0.4 mL/min.

### Data processing and calculations

Reported yield coefficients were always based on mass. Yield coefficients for the xylose phase (Additional files 2 and 4) were calculated for the second phase of the fermentation when glucose was depleted. Carbon balance calculations included metabolite and biomass yields. For the biomass yield a value of 26.4 g/C-mol was utilized [47]. It was further assumed that 1 mol CO_2_ was formed per mol acetate and ethanol. *q*_Glucose_ and *q*_Xylose_ were calculated by plotting glucose and xylose concentration against fermentation time and fitting the concentration decay with a suitable equation. The first derivative of the respective equation, normalized on the CDW, was used to calculate the uptake rate, which is given in g/g_CDW_/h. Similar to previously published studies [22,40], *q*_Xylose_ was observed to decrease with reaction time (Additional file 5). Values of *q*_Xylose_ reported herein are therefore calculated from the initial period of the xylose phase (when glucose was already depleted fully) and represent arithmetic means of at least two determinations made within the first 5 hours of this phase [48]. The courses of *q*_Xylose_ over time are provided in Additional file 5.

## Abbreviations

AN: Anaerobic; ATP: Adenosine triphosphate; CDW: Cell dry weight; DM: Dry mass; FPU: filter paper unit; Glc: Glucose; HCD: High cell density; HPLC: High performance liquid chromatography; IUPAC: International Union of Pure and Applied Chemistry; LCD: Low cell density; MM/M-medium: Mineral medium; NADH: Nicotinamide adenine dinucleotide; NADP: Nicotinamide adenine dinucleotide phosphate; NREL: National Renewable Energy Laboratory; OD600: Optical density at 600 nm; Pep: Peptone; PPP: Pentose phosphate pathway; qXylose/Glucose: Specific xylose/glucose uptake rate; Semi-AN: Semi-anaerobic; SHCF: Separate hydrolysis and co-fermentation; SHF: Separate hydrolysis and fermentation; SSF: Simultaneous saccharification and fermentation; WIS: Water-insoluble content; XDH: Xylitol dehydrogenase; XI: Xylose isomerase; XM-medium: Xylose mineral medium; XR: Xylose reductase; Xyl: Xylose; Xyl/Glc: Xylose/glucose; YEthanol/Glycerol/Xylitol/Acetate: Ethanol/glycerol/xylitol/acetate yield; YPD: Yeast extract peptone dextrose; YX: Yeast extract xylose; μXylose: Maximum specific anaerobic growth rate.

## Competing interests

The authors declare that they have no competing interests.

## Authors’ contributions

VN, SK, MK and BN designed the research. VN and SK planned the experiments. VN, SK, KL, GM and MW performed the experiments and analyzed data. The manuscript was written from contributions of all authors. VN and BN wrote the paper. All authors read and approved the final manuscript.

## Supplementary Material

Additional file 1 **Fermentation of YX media using strains (A) BP10001, (B) IBB10A02 and (C) IBB10B05.** Full diamonds, xylose; empty triangles, glycerol; empty squares, xylitol; empty circles, ethanol; crosses, OD_600_. Click here for file

Additional file 2 Product yields obtained in fermentations of YX media utilizing strains BP1000, IBB10A02 and IBB10B05. Click here for file

Additional file 3 **‘Glucose phase’ of mixed glucose-xylose fermentation in 5% hydrolyzate**_**X**_**.** Depicted are the first 8 h of fermentation using strains **(A)** BP10001, **(B)** IBB10A02 and **(C)** IBB10B05. Full time courses are depicted in Figure 2. Full diamonds, xylose; crosses, glucose; empty triangles, glycerol; empty squares, xylitol; empty circles, ethanol. Click here for file

Additional file 4 **Product yields obtained in the ‘xylose phase’ in fermentations of 5% hydrolyzate**_**X **_**and 15% hydrolyzate utilizing strains BP10001, IBB10A02 and IBB10B05.** Click here for file

Additional file 5 ***q***_**Xylose **_**is decreasing with fermentation time.** Depicted is the *q*_Xylose_ over fermentation time in fermentation of **(A)** YX, **(B)** 5% hydrolyzate_X_ and **(C)** 15% hydrolyzate using strains BP10001 (empty triangles), IBB10A02 (filled squares) and IBB10B05 (filled circles). Click here for file
