# Supplementary figures and images for: Process intensification through microbial strain evolution: mixed glucose-xylose fermentation in wheat straw hydrolyzates by three generations of recombinant Saccharomyces cerevisiae
Source: Biotechnol Biofuels. 2014 Apr 3;7:49. doi: 10.1186/1754-6834-7-49 (PMC4234986; doi:10.1186/1754-6834-7-49)

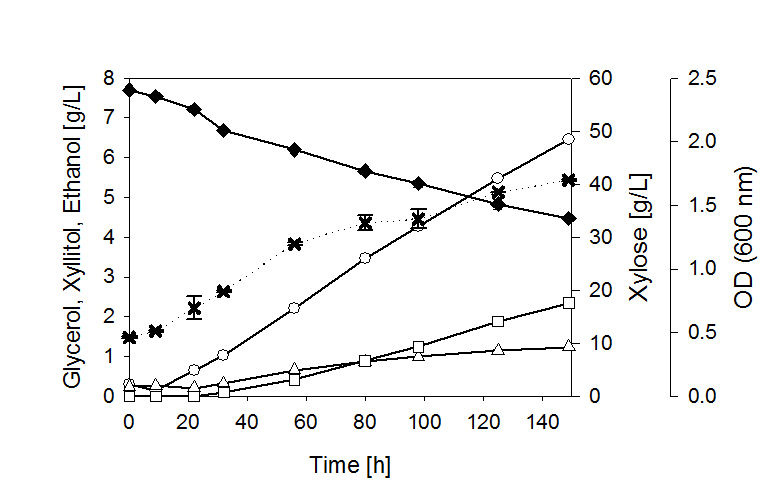

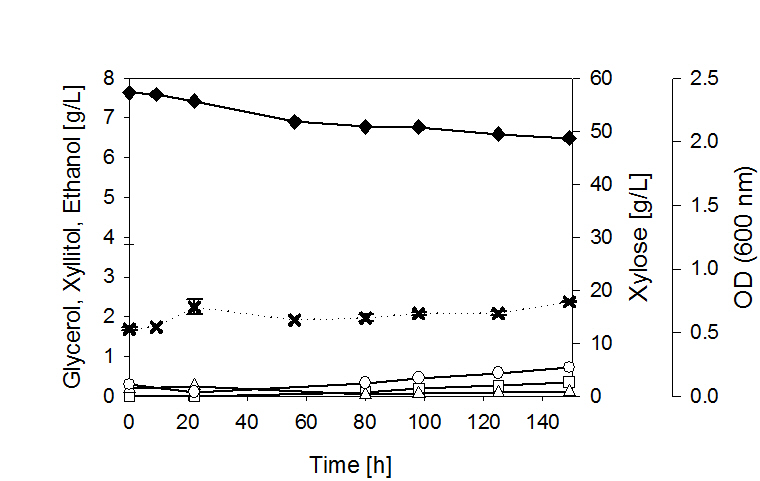


A

B


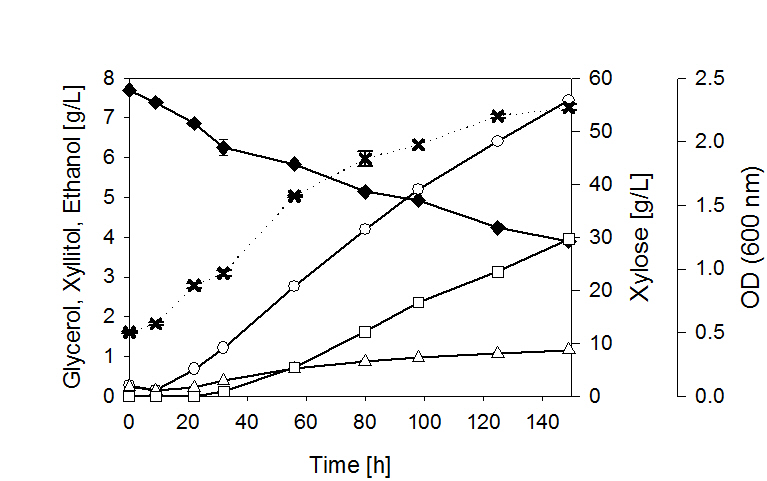


C

Supplement: Additional file 1 — Fermentation of YX media using strains (A) BP10001, (B) IBB10A02 and (C) IBB10B05. Full diamonds, xylose; empty triangles, glycerol; empty squares, xylitol; empty circles, ethanol; crosses, OD600. [file 1754-6834-7-49-S1.docx]

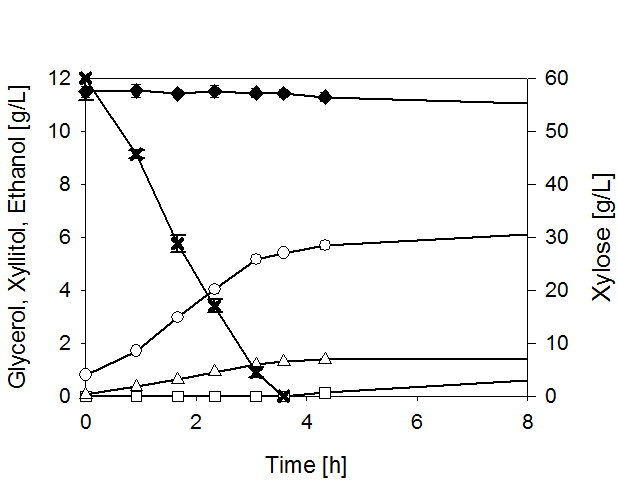

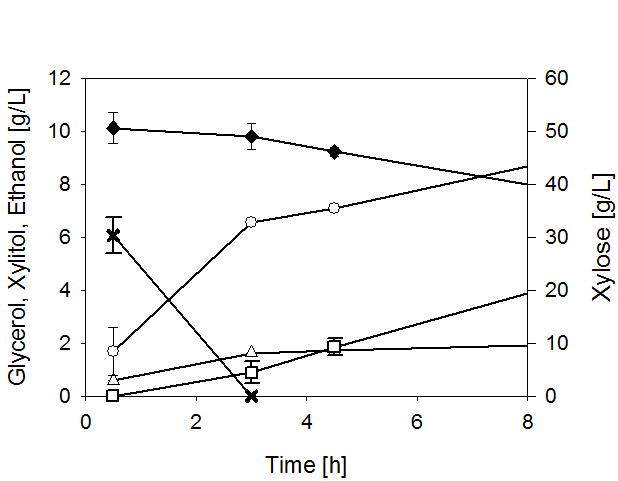


B

A

A

**
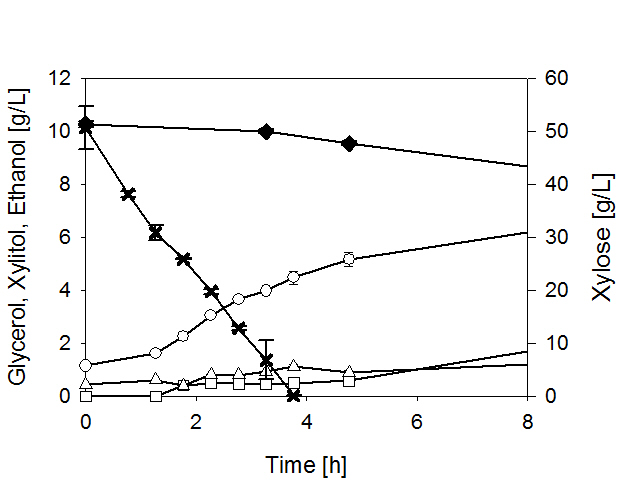
**

C

Supplement: Additional file 3 — ‘Glucose phase’ of mixed glucose-xylose fermentation in 5% hydrolyzateX. Depicted are the first 8 h of fermentation using strains (A) BP10001, (B) IBB10A02 and (C) IBB10B05. Full time courses are depicted in Figure 2. Full diamonds, xylose; crosses, glucose; empty triangles, glycerol; empty squares, xylitol; empty circles, ethanol. [file 1754-6834-7-49-S3.docx]

**
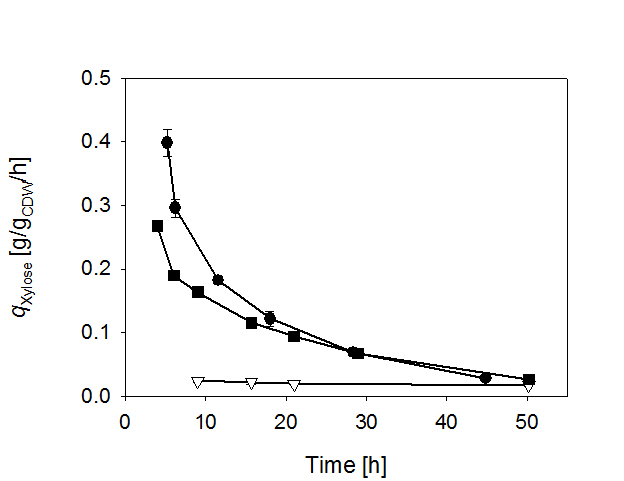

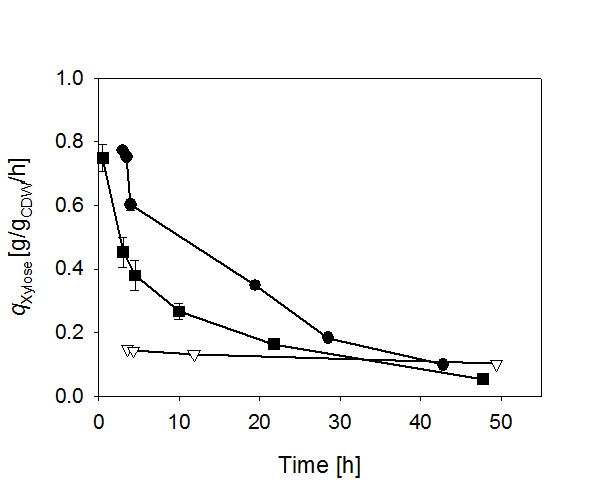

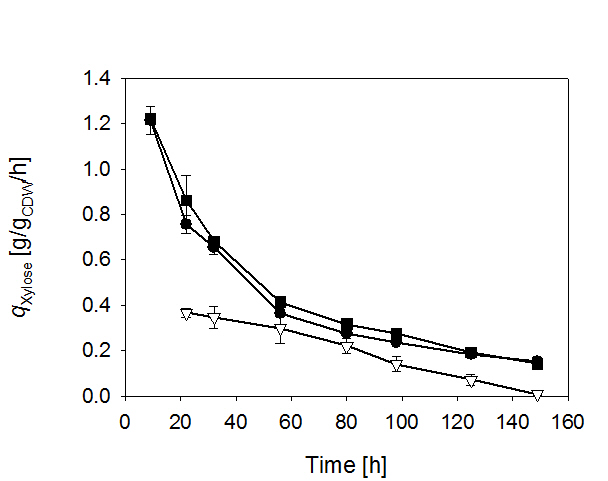
**

C

B

A

Supplement: Additional file 5 — qXylose is decreasing with fermentation time. Depicted is the qXylose over fermentation time in fermentation of (A) YX, (B) 5% hydrolyzateX and (C) 15% hydrolyzate using strains BP10001 (empty triangles), IBB10A02 (filled squares) and IBB10B05 (filled circles). [file 1754-6834-7-49-S5.docx]
